# The triad in current neuroblastoma challenges: Targeting antigens, enhancing effective cytotoxicity and accurate 3D *in vitro* modelling

**DOI:** 10.1016/j.tranon.2024.102176

**Published:** 2024-11-02

**Authors:** Ellen King, Ronja Struck, Olga Piskareva

**Affiliations:** a Cancer Bioengineering Group & Tissue Engineering Research Group (TERG), Department of Anatomy and Regenerative Medicine, RCSI University of Medicine and Health Sciences, Dublin, Ireland; b School of Pharmacy and Biomolecular Sciences, RCSI University of Medicine and Health Sciences, Dublin, Ireland; c Advanced Materials and Bioengineering Research Centre (AMBER), RCSI University of Medicine and Health Sciences and Trinity College Dublin, Dublin, Ireland

**Keywords:** Neuroblastoma, Immunotherapy, Tumour associated antigens, 3D *in vitro* cancer models

## Abstract

Neuroblastoma is an embryonic tumour originating from neural crest cells and accounts for nearly 15 % of all childhood cancer deaths. Despite the implementation of intense multimodal therapy for neuroblastoma, half of the high-risk cohort will relapse with metastatic foci resistant to conventional therapies. There is an urgent need for novel precision medicine approaches to improve patient survival and ensure healthy post-treatment lives for these children.

Immunotherapy holds promise for such therapeutics; however, developing effective options has been disappointing despite decades of research. The immunosuppressive tumour-immune microenvironment presents a significant challenge amplified with low mutational burden in neuroblastoma, even with the new discovered tumour antigens. Innovative, practical, and comprehensive approaches are crucial for designing and testing immunotherapies capable of passing clinical trials. Replacing animal models with physiologically relevant *in vitro* systems will expedite this process and provide new insights into exploitable tumour-immune cell interactions. This review examines this three-pronged approach in neuroblastoma immunotherapy: tumour antigen discovery, immunomodulation, and 3D *in vitro* tumour models, and discusses current and emerging insights into these strategies to address neuroblastoma immunotherapy challenges.

## Highlights

• Current immunotherapeutic approaches for neuroblastoma are insufficient. To improve this, we need to better understand the immunogenicity of the patient, identify an appropriate target antigen and model it in 3D *in vitro* to understand the medication on a deeper level.
• Immunotherapies require innovative approaches based on the advances in adult cancer technologies to make them sufficiently effective in the sparse immune landscape of neuroblastoma.
• Identifying appropriate neoantigens and patient immune status pose great challenges in the development of personalised immunotherapies. Artificial intelligence will step in as a crucial player in tackling those.
• Accurate 3D modelling of tumour-immune interactions is currently underused in understanding those interactions, but holds the key to improving the slow bench-to-bedside pipeline.

## Graphical abstract

**Figure:**
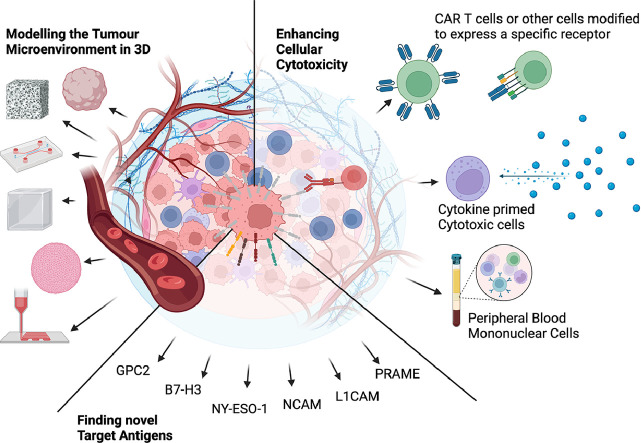

## Introduction

**Fig. 1:**
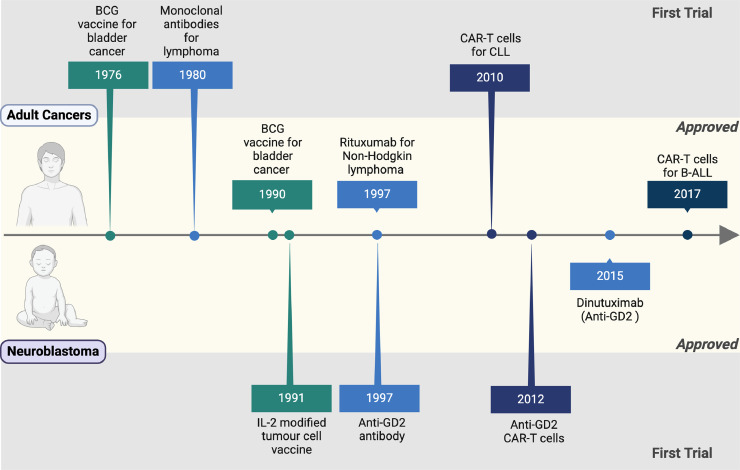
The timeline of immunotherapy development in neuroblastoma. Created with BioRender.com.

Neuroblastoma is an embryonic tumour originating from neural crest cells in the developing foetus or during the early years of life. The aggressiveness of neuroblastoma is driven by many molecular features that contribute to its heterogeneity, including age at diagnosis, tumour localisation and size, histopathologic classification, DNA content, chromosome imbalances and oncogene amplification [[1], [2], [3]]. The addition of multi-modal treatment regimens has boosted the survival probabilities for high-risk neuroblastoma from 15 % to almost 50 % [4]. However, nearly half of all high-risk patients do not respond to first-line therapy, progressing to the maintenance phase of treatment with more novel treatments required to tackle minimal residual disease [5]. Immunotherapy represents a key player in these novel therapeutic interventions though progress in their development, testing and approval remains tiresomely slow (Fig. 1). Partially, this is due to its unique tumour-immune microenvironment with low immunogenicity and a large number of immunomodulatory mechanisms contributing both locally and systemically, which, in turn, leads to low numbers and questionable tumour reactivity of tumour infiltrating lymphocytes impeding successful implementation of immunotherapy [6]. Major challenges to speed up this process lie in the identification of actionable tumour-associated antigens (TAA), overcoming the immunologically “cold” neuroblastoma tumour microenvironment and using novel techniques to test immunotherapeutics *in vitro* limiting the use animal models. This review collates the current and emerging insights into this three-pronged approach to tackle the challenge of neuroblastoma immunotherapy.

## Emerging neuroblastoma immunotherapies

**Fig. 2:**
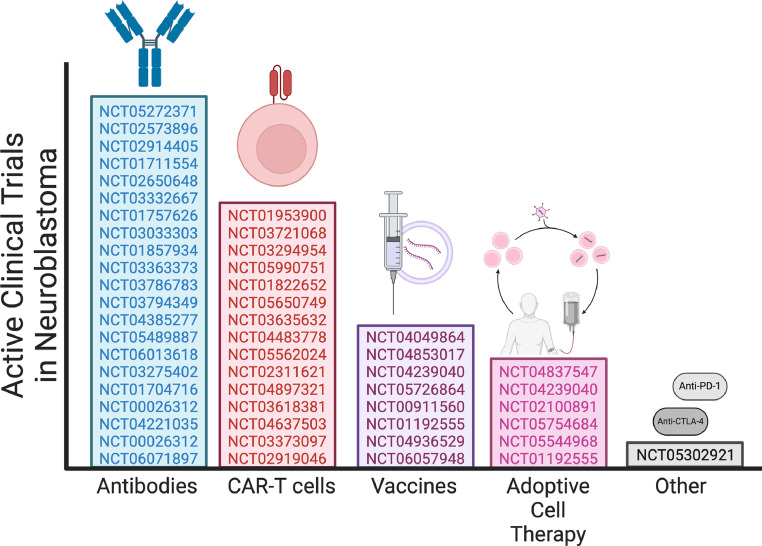
Active trails in neuroblastoma. Created with BioRender.com.

Most active clinical trials for neuroblastoma immunotherapies focus on anti-GD2 monoclonal antibodies directed against the disialoganglioside GD2 (Fig. 2). This is the first TAA with FDA/EMA approved immunotherapy. Other emerging novel options, such as CAR-T cells, vaccines, and adoptive cell therapies, are also under evaluation.

Currently, immunotherapy is given during the maintenance phase of neuroblastoma treatment plans to patients who have already undergone an extremely intensive cytotoxic regimen. Lymphopenia is a common side effect of many chemotherapeutic agents, reducing total T cell counts in patients [7]. This can lead to compromised tumour-specific T-cell responses [8]. Of the survivors, chemotherapy-exposed T cells have been described as having reduced metabolism and lower potential to convert to effector or memory states [9,10]. A study by Das et al. described a mere 36 % *ex vivo* processing “pass rate” of neuroblastoma patient-derived T cells (*n* = 11) pre-treatment. This percentage dropped dramatically to 0 % after only one cycle of chemotherapy. The expansion capacity of CD3+ cells recovered to ∼25 % in the third cycle. However, populations of naïve T cells plummeted to <15 %, replaced by a majority proportion of terminal effector T cells (45 %).

Interestingly, effector and central memory T cell subsets almost doubled over 3 courses of chemotherapy, stabilising at such levels throughout the final 2 rounds [9], therefore highlighting significant phenotypic differences between pre-and post-chemotherapy T cells of neuroblastoma patients. Almost 70 % of cells derived from neuroblastoma patients at diagnosis could not pass the *ex vivo* expansion required for treatments such as CAR-T cell therapy [9]. This difficulty can be potentially overcome by recent advances in iPSC-derived T and NK or NK96 cell technologies tested in pre-clinical and early-phase clinical studies in adult cancers and neuroblastoma [[11], [12], [13]]. Additionally, if chemotherapy spares central and effector memory T cells, other types of immunotherapy, such as vaccination, may represent a more logical route to target the specificities of neuroblastoma.

### CAR cells

First-generation CAR-T cells targeting GD2 have shown some clinical responses in neuroblastoma patients with relatively low toxicity levels. In one study (NCT00085930), 3 of 11 patients treated achieved a complete response; one with metastatic disease regressed entirely within 6 weeks but ultimately relapsed. The other two patients remained disease-free 20 and 16 months post-infusion [14]. Another study (NCT02761915) demonstrated mild neurotoxicity (headache and fever-associated hallucination) in only 2 out of 11 patients treated with GD2 CAR-T cells. However, no clinical responses were observed in any patients [15]. Various rounds of CAR-T cell improvements by different groups are ongoing, as are clinical trials which may bring to light more significant patient responses.

Chimeric-antigen receptor natural killer (CAR-NK) cells are derived from primary NK cells or cell lines modified with an antigen-directed chimeric receptor. Although no neuroblastoma clinical trial is currently assessing this type of immunotherapy, pre-clinical work has shown some significance. CAR-NK cells exhibited cytotoxicity against GD2-expressing neuroblastoma tumour cells and the T-cell inhibitory myeloid-derived suppressor cells (MDSCs) [16,17]. A pre-clinical study developed modified NK cells with the activating receptor NKG2D fused to the cytotoxic ζ-chain of the T-cell receptor (NKG2D.ζ) [16]. In mice, these cells eliminated ∼90 % of intra-tumoural myeloid-derived suppressor cells (MDSCs) and resulted in a 400 % reduction in tumour volume compared to unmodified NK cells, which prolonged survival by a median of 44 days. The same study investigated if pre-infusion with these cells could recruit GD2 CAR-T cells to the tumour site, with a 10.9 ± 0.2-fold increase in recruitment [16]. This data highlights the potential of combination immunotherapies.

### Vaccines

There are currently 8 active clinical trials for neuroblastoma that are investigating a vaccine-related therapeutic (Fig 2); 3 immunostimulatory vaccines (NCT04853017, NCT05726864, NCT04936529), 2 modified neuroblastoma cell vaccines (NCT04239040, NCT01192555), 2 vaccines directed against GD2 (NCT00911560, NCT06057948) and 1 DNA vaccine against personalised tumour antigens (NCT04049864). Critical to any vaccine's design, particularly a therapeutic cancer vaccine, is the antigen on which the vaccine is designed to target. It is no surprise that the active clinical trials and the majority of the completed clinical trials for neuroblastoma are either modified neuroblastoma cells reintroduced to patients or peptide vaccines against GD2, the most researched TAA on neuroblastomas.

A phase I trial investigating a dendritic cell vaccine (NCT01241162) showed disappointing results with associated adverse toxic effects in almost half of all patients, mainly myelosuppression due to side-arm decitabine treatment. This vaccine was prepared by isolating a sample of each patient's DCs from peripheral blood mononuclear cells and subsequently stimulating these cells with neuroblastoma TAA peptide pools. These antigens, MAGE-A1, MAGE- A3 and NY-ESO-1, are known to be over-expressed on neuroblastoma and other cancers [18]. The stimulated DCs are then reintroduced to patients once weekly for two weeks. Only two out of ten patients experienced favourable outcomes; one had a complete response, and one remained disease-free 2 years post-treatment [19]. Of note, these patients experienced only minimal disease at the start of the trial. So, while this trial highlights the feasibility of this vaccine for some patients, a more potent and specific vaccine is required to address the majority of high-risk patients who present not with minimal disease but with highly aggressive tumours.

Another phase I/II trial (NCT00923351) was completed investigating a tumour lysate-pulsed DC vaccine. Of the 30 patients treated, all patients experienced an immune response to vaccination, 12 remain on follow-up and are stable or without evidence of disease, 7 are receiving additional external therapy, and 11 died from progressive disease. To date, no publication with a detailed breakdown of patient responses exists. However, the results above, again, show highly variable responses from patients.

### Adoptive cell therapy with cytokine-producing cells

Based on NK cells' role in the success of anti-GD2 therapy and their potential for recognition of MHC-lacking neuroblastoma cells, these innate immune cells have attracted interest for use as “off-the-shelf” allogeneic cell therapy. This involves extraction of a patientʼs NK cells, stimulation of the cells *ex vivo* using cytokines and re-infusion to the patient. Currently, there are 20 phase I/II clinical trials investigating NK cell infusions with mixed results from those completed [[20], [21], [22]] or active. A clinical trial examined the longitudinal effects of NK cell infusions. Post-chemo-immunotherapy infusion with NK cells stimulated with IL-2/IL-15 *ex vivo* improved NK cell cytotoxicity with a range of 4.32–21.56 % increase across 6 patients [21]. While this therapeutic seems well tolerated in neuroblastoma patients, the direct contribution of NK cell infusions to survival outcomes remains to be seen.

## Emerging neuroblastoma-associated antigens

Most immunotherapies for neuroblastoma have focused on the disialoganglioside GD2, based on the high density of over-expression on tumour cells and low levels of expression on healthy cells. However, significant neural toxicities have been associated with this therapy due to the expression of GD2 on nerve cells [23]. To combat this, many groups have expanded on novel immunotherapeutic target discovery with antigens such as glypican-2 (GPC2), New York oesophageal squamous cell carcinoma 1 (NY-ESO-1), B7-H3 (also known as CD276), neural cell adhesion molecule (NCAM)(also known as CD56), L1 cell adhesion molecule (L1CAM) (also known as CD171), preferentially expressed in melanoma antigen (PRAME) and programmed death-ligand 1 (PD-L1) (also known as CD274) showing more promise in the neuroblastoma field (reviewed extensively in [4,6]).

### GPC2

GPC2 is membrane-expressed proteoglycan and part of a family of 6 proteins with involvement in wingless (Wnt), hedgehog (Hh), fibroblast growth factor (FGF), and bone morphogenetic protein (BMP) signalling pathways [24]. GPC2 is over-expressed on neuroblastoma, as well as a variety of other cancers such as small-cell lung cancer, osteosarcoma and Ewing sarcoma [[25], [26], [27]]. Health tissue expression of this antigen is highly restricted, with low levels of GPC2 found mainly in the testes [25]. This poses GPC2 as a promising immunotherapeutic target, with multiple groups showing anti-neuroblastoma activity in mice treated with CAR-T cells with no significant associated toxicities [25,28,29] and clinical trial underway [NCT05650749]. Approximately 40 % of neuroblastoma patients experience chromosome 7q gain. Given that GPC2 is located on chromosome 7q22.1, therapeutics designed to target this antigen should not be limited by any loss of expression due to genetic abnormalities. A subset of patients with this genetic background could benefit greatly from GPC2-directed therapy.

### NY-ESO-1

NY-ESO-1, located on chromosome Xq28, is a cancer-testis antigen with restricted expression in germ cells [30]. Involvement of NY-ESO-1 in cell cycle progression, cell differentiation, apoptosis and germ cell self-renewal has been suggested [31,32]. This antigen is over-expressed to varying degrees in several cancers, and the restricted expression presents another viable option for immunotherapeutic targeting [33,34]. CAR-T cells directed against NY-ESO-1 significantly delayed localised and disseminated neuroblastoma progression in mice and improved survival. Compared to negative controls, which saw significant tumour progression at day 10, tumours treated with CAR-T cells did not experience considerable progression until between days 25 and 30 (*p* < 0.001). A median survival of 42 days in CAR-T cell-treated mice contrasted sharply to just 17 days for PBS-treated mice [35]. Additionally, a peptide vaccine against this antigen could stimulate both humoral and cellular immune responses in three neuroblastoma patients; however, no survival or anti-tumour activity data has been reported for this small trial. The frequency of NY-ESO-1-specific CD8+ *T* cells rose from undetectable levels pre-vaccination to 0.35 % - 0.82 % after 3 immunisations [33].

### B7-H3

B7-H3 (CD276), located on chromosome 15, is an immune checkpoint protein over-expressed on multiple malignancies including neuroblastoma [36]. B7-H3 works in a pro-tumorigenic fashion from two angles. Firstly, B7-H3 expressed on cancer cells and healthy normal and immune cell types can bind lymphocytes (via a currently unknown receptor), inducing a more regulatory, tumour-promoting phenotype. Secondly, B7-H3 has been implicated in neuroblastoma proliferation, cell cycle arrest and drug resistance [37]. Targeting this antigen in the clinic has progressed to 5 trials investigating B7-H3-directed monoclonal antibodies and 6 focused on CAR-T cells with no published results. The pre-clinical work behind this has shown anti-neuroblastoma activity with both immunotherapeutics (reviewed extensively by[37]).

### NCAM

NCAM (CD56), located on chromosome 11q23, is a glycoprotein expressed on neurons, glia, skeletal muscle, and neuroblastoma tumours [38]. Expression of this antigen correlates with metastatic capacity and stemness in cancer cells. A pre-clinical study investigating two antibody-drug conjugates against NCAM has described potent cytotoxicity *in vitro*. Two of the four neuroblastoma cell lines tested were responsive to ADC treatment after four days, while the others responded by day 6. They found ADC IC50 values of 0.16 and 0.19 pM for SK-N-FI and SK-N-AS cells, respectively. In comparison, an isotype control ADC had detectable cytotoxicity in the same cell lines at >1000 times these concentrations [39]. However, the location of this gene on chromosome 11 could prevent treatment efficacy in the subset of neuroblastoma tumours with 11q loss, approximately 35–45 % of the neuroblastoma patient population[40].

### L1CAM

L1CAM (CD171), a glycoprotein encoded by a gene located on the X chromosome, is part of the family of neural adhesion molecules and has been studied in relation to novel cancer antigens. This protein is involved in tumour cell differentiation, growth, migration and invasion [[41], [42], [43]]. CAR-T cells against L1CAM developed from cells directly isolated from 4 neuroblastoma patients have shown anti-tumour reactivity *in vitro* and in immunocompromised mice. All four CD8+ CAR-T cell products exhibited lysis of CD171+ cells *in vitro* with between 10 % and 20 % increase in lytic activity compared to that against CD171- cells. *In vivo*, these 4 patient-derived CAR-T cell products induced tumour regression on day 5, with a reduction in baseline levels on day 15 [44]. The location of *CD171* on chromosome X does not limit a subsection of the neuroblastoma population with no known genetic aberrations on this chromosome associated with neuroblastoma.

### PRAME

PRAME, another member of the cancer testis antigen family, is expressed by multiple cancers and is considered a potential immunotherapeutic target. While this antigen is yet to be clinically evaluated, pre-clinical analysis has identified PRAME expression in 93 % of primary and 100 % of advanced neuroblastoma patient tumour samples. Increased expression of this protein is associated with tumour stage (high expression found in 80 % of stage 4 neuroblastoma tumours) and age of the patient at diagnosis (higher expression associated with higher age at diagnosis, *p* < 0.01) [45]. *PRAME* is located at chromosome 22q11.22, with no known aberrations in neuroblastoma and, therefore, a potentially significant site to target with immunotherapeutics.

### PD-L1

PD-L1, located on chromosome 9p24.1, is a transmembrane protein that expressed on the surface of some antigen presenting cells and some tumour cells, including neuroblastoma. Its notable therapeutic efficacy lies in blockade of PD-1/PD- L1 interaction by restoring T cell reactivity [46]. PD-L1 expression in neuroblastoma samples returns conflicting results on its detection with IHC and prognostic significance (extensively reviewed in[6]). Nevertheless, clinical trials targeting the PD1-PD-L1 axe are ongoing with Pembrolizumab (NCT02332668), anti-PD1 Nivolumab and/or anti-CTLA4 Ipilimumab (NCT04500548, NCT02304458, NCT03838042, NCT02914405, NCT04412408, NCT01445379) that will provide a robust evidence of their potential efficacy.

## Neuroblastoma immunomodulation

Growing evidence supports the notion that non-specific biological or chemical immunomodulating agents can influence the anti-tumour immune response. Through various downstream mechanisms such as immunogenic tumour cell death, tumour antigen release and subsequent T cell activation, host recognition of tumour cells can be boosted above baseline.

### Biological immunomodulating agents

In neuroblastoma, biological immunomodulating agents are represented by cytokines. Cytokine therapy, mainly interleukin-2 (IL-2) and granulocyte-macrophage colony-stimulating factor (GM-CSF), is used in combination with anti-GD2 antibodies (extensively reviewed by[47]). Using both immunotherapies simultaneously has improved the efficacy of anti-GD2 therapies through increased antibody-dependent cell-mediated cytotoxicity. Almost 20 clinical trials investigating this combination have been completed over the past decade with substantial anti-tumour activity while highlighting the need for close monitoring for adverse effects when treating via cytokines [47]. Cytokine therapy is known for stimulating toxicities in patients across various cancers [48]. Notably, recent results of a neuroblastoma clinical trial (NCT00026312) have shown that 71 % of patients treated with this combination plus isotretinoin were still alive 5 years later [49,50]. While 61 % of patients had no evidence of tumour growth, ∼40 % of patients experienced progression in this trial, and ∼30 % still died from this disease. These results highlight potential benefits of a personalised approach to immunotherapies but further advances are required.

### Chemical immunomodulating agents

On the other hand, traditional chemotherapy can decrease populations of immunosuppressive regulatory T-cells (Tregs) or modulate their function [45,46]. Several chemical drugs are in clinical trials for neuroblastoma with immunomodulating potential (Busulfan, Gemcitabine [[51], [52], [53], [54]], Lenalidomide [55,56], Paclitaxel [[57], [58], [59]], Vincristine, Vorinostat [60,61], Etinostat [62]. Regardless of their original mechanism of action, two of them demonstrated immune cell modulation *in vivo*: vorinostat, an epigenetic drug, and lenalidomide, an immunomodulatory drug with potent antineoplastic, anti-angiogenic, and anti-inflammatory properties.

Epigenetic drugs alter gene transcription by upregulating, downregulating, or silencing genes completely through DNA- and histone-methylation and acetylation. In tumour cells, such aberrant regulation can upregulate TAA expression on the cell surface, enabling the host immune system to attack tumour cells. Studies have demonstrated that vorinostat increased the expression of GD2, the primary target for immunotherapeutic monoclonal antibodies, and shifted a suppressive tumour microenvironment (TME) to a permissive TME for tumour-directed antibody therapy [60,61]. Vorinostat increased the number of macrophage effector cells expressing high Fc-receptors (FcR) levels and decreased the number and function of myeloid-derived suppressor cells (MDSC).

Another immune-modulating drug, lenalidomide, increased GD2 expression in NLF tumours, which had relatively low ganglioside expression compared to most primary NB [56] and decreased NK cell infiltration. Co-treatment with lenalidomide and ch14.18 suppressed neuroblastoma tumour growth in NOD/SCID mice through activation of NK cells and prevented their suppression by IL-6 and TGFβ1 in the microenvironment. The authors suggested that lenalidomide inhibited STAT3 for IL-6 and SMAD2/3 for TGFβ1.

We do not know how and what doses of chemical agents have a modulating effect on the immune system of patients with neuroblastoma rather than entirely vanishing immune cells together with cancer cells. Preclinical studies similar to [60,61] combined with a systematic evaluation and meta-analysis of the drug immunomodulation could have filled that gap and helped to review current treatment protocols. However, other emerging novel immune-modulating agents could also be considered, such as a small-molecule agonist (ADH-503) [63].

## Modelling neuroblastoma-immune interactions in 3D *in vitro*

**Fig. 3:**
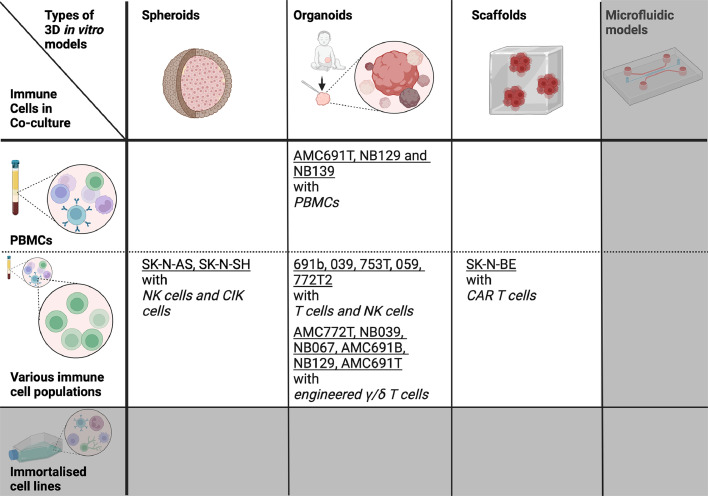
Current 3D *in vitro* models of investigating tumour immune-interactions in co-cultures of neuroblastoma (underlined) and immune cells (cursive). The immune cells can be derived from immortalised cell lines or Peripheral Blood Mononuclear Cells (PBMCs) isolated from donor blood. The grey area indicates that no data published at the time of review submission. Created with BioRender.com.

Replication of the tumour-immune landscape *in vitro* is crucial with the recent rise of immunotherapy development. The 3D *in vitro* cancer concepts and tools have been amply reviewed for neuroblastoma (e.g. [64].). We conducted a comprehensive PubMed search for the 3D *in vitro* models of neuroblastoma investigating a tumour-immune interaction or immune evasion mechanisms. The search returned a handful of papers published in the past 5 years, highlighting the novelty of this field (Fig 3).

Co-culture of cancer cells with immune cells represents a natural increase in 3D model complexity. While possible to include immortalised cell lines, all models discussed here, were created using immune cells isolated from donor blood, either PBMCs [65,66] or, more specifically, T or NK cells only [65,[67], [68], [69]] (Fig 3). To improve cytotoxicity, they were primed with cytokines [65,67] or modified to express a specific γ/δ-T cell receptor [68] or a chimeric antigen receptor [69].

### Immunotherapeutics and antibody-drug conjugates

Immunotherapeutics are the most common investigation using the 3D *in vitro* models and focusing on modified T cells [[67], [68], [69]]. Grunewald et al. concluded their CAR T cells demonstrated physiological relevance better in 3D than 2D, where cytotoxicity was less pronounced. Almost 100 % cell lysis was reported in 2D co-cultures, with only around 40 % killing in 3D (*p* < 0.05) [69]. Interestingly, as measured by CD25 and CD137 expression, T cell activation showed an opposite trend, with expression increasing about 30 % from 2D to 3D.

Strijker et al. found their γδ-T cells, engineered from αβ-T cells, produced IFN-γ in co-culture with half of the tested patient-derived organoids of up to 200 pg/mL as opposed to untransduced αβ-T cells which did not trigger IFN-γ production[68]. While this T cell activation was found to be independent of MHC I expression, Pamidronate treatment increased lysis by up to 6-fold.

Cornel et al. found that (PRAME)-reactive tumour-specific T cells increase patient-derived neuroblastoma organoid killing by about 100 %; 40 % and 70 % after pre-treatment with IFN-γ (*p* < 0.0001), IFN-α (*p* < 0.0001) and immunomodulator entinostat, respectively (*p* < 0.0001)[67]. On the other hand, healthy-donor natural killer (NK) cells increased their killing only in response to IFN-α and entinostat by about 10 % and 30 %, respectively.

Instead of T cells, Heinze et al. [65]. and Kholosy et al. [66]. focussed on NK and Cytokine-induced killer (CIK) cells or collective peripheral blood mononuclear cells (PBMCs), respectively. The former found both cell types to be effective in tumour cell eradication at various effector:target (E:T) ratios, with NK cells outperforming CIKs in the short-term killing. Kholosy et al. used PBMCs to validate their model with the FDA-approved anti-GD2 immunotherapeutic [66].

Finally, using the antibody-drug conjugate vobramitamab duocarmazine rather than an immunotherapy, Brignole et al. found a dose-dependent viability reduction in neuroblastoma spheroids[70].

### Effector: Target ratios

Co-culture experiments are particularly relevant for our understanding of molecular mechanisms triggering tumour cell recognition by immune cells. Different effector: target cell ratios (E:T) may help to shed light on the required numbers of effector cells, as different ratios can result in very different degrees of cancer cell eradication. Neuroblastoma cell lysis of NK cells via europium release was tested in 4 different E:Ts ranging from 10:1 to 0.5:1 in different types of medium [65]. Degree of lysis inversely correlated to E:T with up to 94.92 % lysis at 10:1 and less than 20 % at an E:T of 0.5:1. This highlights the crucial effect a chosen E:T has on the apparent effectiveness of a drug, begging the question of the most physiologically relevant E:T. Other researchers used only single E:Ts in their immunotherapeutic studies. Strijker et al. did not find a clear trend with E:Ts of 0.3:1, 1:1, and 3:1 using engineered γδ- T cells [68]. They instead observed 10 µM Pamidronate to increase cytotoxicity up to 6-fold. Cornel et al. chose 1:1 [67], and Grunewald et al. picked 5:1, which was physiologically reduced to 1:10 through limited T-cell infiltration [69]. Kholosy et al. used 20:1 [66], the only ones using healthy donor PBMCs as effector cells. Thus, more immune cell types, including ones with lower or no efficacy, interacted within a single 3D cancer model, resulting in more variables to assess. However, the question remains: what E:T is physiologically relevant? As not otherwise stated, the above studies used immune cells from healthy donors, presumably adults. Using healthy donor effector cells does not reflect the actual scenario in modelling immunotherapeutics *in vitro and in vivo*, taking into consideration marked differences in the expression profiles of PBMCs from healthy donors and cancer patients of different stages [71], with expression profiles even potentially predicting relapse [72]. Notably, PBMCs are typically depleted or exhausted in cancer patients[9]. Similarly, adult and paediatric PBMCs or neonatal cord blood differ in cell type abundance [73] and function [74]. While these facts highlight current limitations in 3D cancer models, the published sc-RNS profiling of various immune cell population can bridge the gap by selecting clinically relevant immune cells and their sources [75]. This in turn, will facilitate the evidence-based move from the lab to the clinic for immunotherapies.

### Receptor expression

Alongside cytotoxic investigations, receptor expression was a commonly investigated immune interaction feature [65,67,68]. Specifically, expression levels of major histocompatibility I (MHC I) are a protein expressed on the membrane of all cells in an organism and used to present fragments of proteins within the cell to the immune system. Low MHC I expression of tumour cells is a well-known mechanism of immune evasion [76]. Cornel et al. found MHC I expression increased by exposure to cytokines IFN-α and IFN-γ and even more by exposure to cytokines and entinostat (up to 10-fold chance at *p* < 0.01 to *p* < 0.0001)[67]. Both Heinze et al. and Strijker et al. related the cell inherent MHC I expression profiles to their investigations [65,68]. While Strijker et al. reported their cell-based immunotherapeutic works in an MHC I independent manner [68], Heinze et al. found slightly greater killing efficacy of NK cells against the MHC I low SK-N-SH spheroids than the MHC I high SKN-AS spheroids [65].

### 2D vs 3D *in vitro* co-culture

From the early days of 3D *in vitro* modelling, some marked differences in cell behaviour between 2D and 3D and compared to *in vivo*, such as drug tolerances, have been reported [77,78]. Two independent studies compared their 3D data to 2D cell culture conditions [69,70]. Brignole et al. found the dose-dependent viability reductions in response to the antibody-drug conjugate, vobramitamab duocarmazine confirmed in 2D [70]. However, higher drug concentrations were required in 3D to reach a 50 % reduction in cell viability. Similarly, Grunewald et al. found CAR-T cell-mediated killing more effective in 2D but more strongly activated in 3D as measured by CD25 (depending on CAR T-cell type up to *p* ≤ 0.01) and CD137 (*p* ≤ 0.5) expression [69].

Out of all papers discussed in this section, Brignole et al. compared their 3D *in vitro* model to *in vivo* data. They found the reduced cancer cell viability found in 3D *in vitro* in response to vobramitamab duocarmazine translated to extended survival times in 5 out of 6 mouse models of 7 to 49 days [70].

In comparison with *in vivo* models, 3D *in vitro* models provide a system to experiment with the extracellular matrix and how different stiffnesses affect immune and tumour cell behaviour (Table 1). In a hydrogel model of human adenocarcinoma for example it has been found that stiffness and macrophage phenotype jointly cause a more invasive tumour phenotype, highlighting the importance of 3D models over *in vivo* models in investigating cellular cross talk [79].

**Table 1:** Comparison of 2D, 3D cell culture and *in vivo* models of neuroblastoma.

	2D cell culture	Attachment independent 3D models (spheroids and organoids)	Attachment dependent 3D models (those including ECM)	*In vivo* animal models
Cost	€	€	€€ - €€€	€€€
Ease of Acquisition	Easy	Easy to very difficult	Easy to Intermediate	Difficult, considering the ethical approvals and facilities required
Specialised equipment required	None	None	**Scaffolds**: freeze dryer or electrospinning apparatus **Tumour-on-a-chip**: Photolithography Setup or Etching Equipment or Soft Lithography Tools or a 3D Printer for the fabrication of the chip and a rotary culture system to maintain a dynamic culture	Animal housing facilities, surgical and injection equipment, imaging, monitoring and data collection equipment
Biological Complexity	Low	Intermediate	Intermediate	High
Technical complexity	Low	Intermediate	Intermediate	High
Human Relevance	Low	Intermediate	Intermediate to High	High
Immune system interactions	Simple immune cell – cancer cell interactions	Both simple and complex immune cell – cancer cell interactions and invasion through 3D culture	Both simple and complex immune cells – cancer cell interactions and invasion through 3D culture and extracellular matrix. Perfusion is possible	Typical, immunodeficient models otherwise murine immune system
Human immune system targets	Yes	Yes	Yes	Only in immunodeficient xenografts
Modelling matrix interactions	No	No	Yes	Yes
Labour intensiveness	Low	Low-Intermediate	Intermediate	High

## Conclusions and future directions

Childhood cancers are relatively uncommon, with a low mutational burden compared to adult cancers. While immunotherapy holds great promise, it still faces many challenges in treating neuroblastoma. Probably the most significant two are the selection of neoantigens and the immune status of a cancer patient. Neuroblastoma patients undergo intensive, immune-depleting treatments, prior to the introduction of immunotherapy. Patients that do progress to immunotherapeutic approaches are often those with the most aggressive disease and under-performing anti-tumour immunity. A major addition to the field would be more complex understanding of the neuroblastoma-immune microenvironment and indeed, the propensity for immune activation in treated patients. Multiantigen approaches combined with immunomodulators can provide better recognition of tumour cells and its heterogeneity by the patient immune system.

To progress multiantigen approaches actionable TAAs are a must. This is, where Artificial Intelligence (AI) comes at a play to interrogate vast datasets in automating quantitative fashion to predict candidates for immunotherapy [80]. By harnessing and scrutinising biomedical data, including omics, radiology, pathology, and clinical data, AI algorithms bolster their capacity to execute the five steps of TAA prediction. This includes somatic mutation calling, MHC typing, assessment of peptide-HLA binding affinity, TCR-pMHC binding prediction, and prediction of the immunogenicity of TAA candidates. Paired with sc-RNAseq datasets of immune status of patients with neuroblastoma, AI can model potential responses to immunotherapy *in silico.*

Within the realm of 3D tumour-immune models, this review primarily focuses on a few relevant examples related to neuroblastoma. By leveraging bio- and tissue engineering advances, we can meticulously reconstruct the interactions between neuroblastoma and diverse immune cells. This approach offers profound insights into the cell-to-cell interactions that contribute to the 'cold' immune microenvironment of neuroblastoma (Table 1). The future holds promise for advancements in microfluidic models that explore the interactions between tumour and immune cells extravasating from synthetic blood vessels to simulate immune invasion. This aspect of the immune response is a significant limitation of current *in vitro* models when compared to their *in vivo* counterparts.

Enhanced with AI, these 3D models can also help study the effects of immunomodulating agents on TAA expression and immune cell activation, thus accelerating the drug discovery pipeline for personalised immunotherapies.

## Disclosure

The funders had no role in study design, data collection and analysis, decision to publish, or preparation of the manuscript. The authors declare that they have no conflict of interest.

## Declaration of generative AI and AI-assisted technologies in the writing process

During the preparation of this work the authors used Grammarly in order to improve language and readability, with caution. After using this tool, the authors reviewed and edited the content as needed and took full responsibility for the content of the publication.

## CRediT authorship contribution statement

**Ellen King:** Writing – review & editing, Writing – original draft, Visualization, Conceptualization. **Ronja Struck:** Writing – review & editing, Writing – original draft, Visualization, Funding acquisition, Conceptualization. **Olga Piskareva:** Writing – review & editing, Writing – original draft, Supervision, Resources, Project administration, Funding acquisition, Conceptualization.

## Declaration of competing interest

On behalf of my co-authors, I am submitting the enclosed original review article for consideration for publication in *Translational Oncology*. It is not under consideration for publication elsewhere, nor has it been published in whole or in part elsewhere. All the authors were fully involved in the manuscript preparation and agreed on its submission to *Translational Oncology*. None of the authors have any conflicts of interest.

## References

[1] Riley R.D., Heney D., Jones D.R., Sutton A.J., Lambert P.C., Abrams K.R. (2004). A systematic review of molecular and biological tumor markers in neuroblastoma. Clin. Cancer Res..

[2] Bagatell R., DuBois S.G., Naranjo A., Belle J., Goldsmith K.C., Park J.R. (2023). Children's oncology Group's 2023 blueprint for research: neuroblastoma. Pediatr. Blood Cancer.

[3] Liang W.H., Federico S.M., London W.B., Naranjo A., Irwin M.S., Volchenboum S.L. (2020). Tailoring therapy for children with neuroblastoma on the basis of risk group classification: past, present, and future. JCO Clin. Cancer Inform..

[4] Anderson J., Majzner R.G., Sondel P.M. (2022). Immunotherapy of neuroblastoma: facts and hopes. Clin. Cancer Res..

[5] Segura M.F., Soriano A., Roma J., Piskareva O., Jiménez C., Boloix A. (2022). Methodological advances in the discovery of novel neuroblastoma therapeutics. Expert Opin. Drug Discov..

[6] Wienke J., Dierselhuis M.P., Tytgat G.A.M., Künkele A., Nierkens S., Molenaar J.J. (2021). The immune landscape of neuroblastoma: challenges and opportunities for novel therapeutic strategies in pediatric oncology. Eur. J. Cancer.

[7] Lee Y.T., Tan Y.J., Oon C.E. (2018). Molecular targeted therapy: treating cancer with specificity. Eur. J. Pharmacol..

[8] Sharma A., Jasrotia S., Kumar A. (2024). Effects of chemotherapy on the immune system: implications for cancer treatment and patient outcomes. Naunyn Schmiedebergs Arch. Pharmacol..

[9] Das R.K., Vernau L., Grupp S.A., Barrett D.M. (2019). Naïve T-cell deficits at diagnosis and after chemotherapy impair cell therapy potential in pediatric cancers. Cancer Discov..

[10] Buck M.D., O'Sullivan D., Klein Geltink R.I., Curtis J.D., Chang C.-H., Sanin D.E. (2016). Mitochondrial dynamics controls T cell fate through metabolic programming. Cell.

[11] Patel S.J., Yamauchi T., Ito F. (2019). Induced pluripotent stem cell-derived T cells for cancer immunotherapy. Surg. Oncol. Clin. N. Am..

[12] Maddineni S., Silberstein J.L., Sunwoo J.B. (2022). Emerging NK cell therapies for cancer and the promise of next generation engineering of iPSC-derived NK cells. J. Immunother. Cancer.

[13] McNerney K.O., Karageorgos S.A., Hogarty M.D., Bassiri H. (2020). Enhancing neuroblastoma immunotherapies by engaging iNKT and NK cells. Front. Immunol..

[14] Louis C.U., Savoldo B., Dotti G., Pule M., Yvon E., Myers G.D. (2011). Antitumor activity and long-term fate of chimeric antigen receptor–positive T cells in patients with neuroblastoma. Blood.

[15] Straathof K., Flutter B., Wallace R., Jain N., Loka T., Depani S. (2020). Antitumor activity without on-target off-tumor toxicity of GD2–chimeric antigen receptor T cells in patients with neuroblastoma. Sci. Transl. Med..

[16] Parihar R., Rivas C., Huynh M., Omer B., Lapteva N., Metelitsa L.S. (2019). NK cells expressing a chimeric activating receptor eliminate MDSCs and rescue impaired CAR-T cell activity against solid tumors. Cancer Immunol. Res..

[17] Esser R., Müller T., Stefes D., Kloess S., Seidel D., Gillies S.D. (2012). NK cells engineered to express a GD 2 -specific antigen receptor display built-in ADCC-like activity against tumour cells of neuroectodermal origin. J. Cell Mol. Med..

[18] Bao L., Dunham K., Lucas K, MAGE-A1 (2011). MAGE-A3, and NY-ESO-1 can be upregulated on neuroblastoma cells to facilitate cytotoxic T lymphocyte-mediated tumor cell killing. Cancer Immunol. Immunother..

[19] Krishnadas D.K., Shusterman S., Bai F., Diller L., Sullivan J.E., Cheerva A.C. (2015). A phase I trial combining decitabine/dendritic cell vaccine targeting MAGE-A1, MAGE-A3 and NY-ESO-1 for children with relapsed or therapy-refractory neuroblastoma and sarcoma. Cancer Immunol. Immunother..

[20] Talleur A.C., Triplett B.M., Federico S., Mamcarz E., Janssen W., Wu J. (2017). Consolidation therapy for newly diagnosed pediatric patients with high-risk neuroblastoma using busulfan/melphalan, autologous hematopoietic cell transplantation, anti-GD2 antibody, granulocyte-macrophage colony–stimulating factor, interleukin-2, and haploidentical natural killer cells. Biol. Blood Marrow Transplant.

[21] Nguyen R., Sahr N., Sykes A., McCarville M.B., Federico S.M., Sooter A. (2020). Longitudinal NK cell kinetics and cytotoxicity in children with neuroblastoma enrolled in a clinical phase II trial. J. Immunother. Cancer.

[22] Modak S., Le Luduec J.-B., Cheung I.Y., Goldman D.A., Ostrovnaya I., Doubrovina E. (2018). Adoptive immunotherapy with haploidentical natural killer cells and Anti-GD2 monoclonal antibody m3F8 for resistant neuroblastoma: results of a phase I study. Oncoimmunology.

[23] Blom T., Lurvink R., Aleven L., Mensink M., Wolfs T., Dierselhuis M. (2021). Treatment-related toxicities during anti-GD2 immunotherapy in high-risk neuroblastoma patients. Front. Oncol..

[24] Filmus J., Capurro M., Rast J. Glypicans. Genome Biol.[Internet]. 2008;9(5):224. Available from: http://genomebiology.biomedcentral.com/articles/10.1186/gb-2008-9-5-224. 10.1186/gb-2008-9-5-224 PMC2441458 18505598

[25] Bosse K.R., Raman P., Zhu Z., Lane M., Martinez D., Heitzeneder S. (2017). Identification of GPC2 as an oncoprotein and candidate immunotherapeutic target in high-risk neuroblastoma. Cancer Cell.

[26] Raman S., Buongervino S.N., Lane M.V, Zhelev D.V, Zhu Z., Cui H. (2021). A GPC2 antibody-drug conjugate is efficacious against neuroblastoma and small-cell lung cancer via binding a conformational epitope. Cell Rep. Med..

[27] Chen G., Luo D., Zhong N., Li D., Zheng J., Liao H. (2022). GPC2 is a potential diagnostic, immunological, and prognostic biomarker in pan-cancer. Front. Immunol..

[28] Heitzeneder S., Bosse K.R., Zhu Z., Zhelev D., Majzner R.G., Radosevich M.T. (2022). GPC2-CAR T cells tuned for low antigen density mediate potent activity against neuroblastoma without toxicity. Cancer Cell.

[29] Li N., Torres M.B., Spetz M.R., Wang R., Peng L., Tian M. (2021). CAR T cells targeting tumor-associated exons of glypican 2 regress neuroblastoma in mice. Cell Rep. Med..

[30] Satie A.-P., Rajpert-De Meyts E., Spagnoli G.C., Henno S., Olivo L., Jacobsen G.K. (2002). The cancer-testis gene, NY-ESO-1, is expressed in normal fetal and adult testes and in spermatocytic seminomas and testicular carcinoma in situ. Lab Investig..

[31] Kisseleva-Romanova E., Lopreiato R., Baudin-Baillieu A., Rousselle J.-C., Ilan L., Hofmann K. (2006). Yeast homolog of a cancer-testis antigen defines a new transcription complex. EMBO J..

[32] Cronwright G., Le Blanc K., Götherström C., Darcy P., Ehnman M., Brodin B (2005). Cancer/testis antigen expression in human mesenchymal stem cells: down-regulation of SSX impairs cell migration and matrix metalloproteinase 2 expression. Cancer Res..

[33] Camisaschi C., Renne S.L., Beretta V., Rini F., Spagnuolo R.D., Tuccitto A. (2018). Immune landscape and *in vivo* immunogenicity of NY-ESO-1 tumor antigen in advanced neuroblastoma patients. BMC Cancer.

[34] Thomas R., Al-Khadairi G., Roelands J., Hendrickx W., Dermime S., Bedognetti D. (2018). NY-ESO-1 based immunotherapy of cancer: current perspectives. Front. Immunol..

[35] Singh N., Kulikovskaya I., Barrett D.M., Binder-Scholl G., Jakobsen B., Martinez D. (2016). T cells targeting NY-ESO-1 demonstrate efficacy against disseminated neuroblastoma. Oncoimmunology.

[36] Getu A.A., Tigabu A., Zhou M., Lu J., Fodstad Ø., Tan M (2023). New frontiers in immune checkpoint B7-H3 (CD276) research and drug development. Mol. Cancer.

[37] Pulido R., Nunes-Xavier C.E (2023). Hopes on immunotherapy targeting B7-H3 in neuroblastoma. Transl. Oncol..

[38] Heinly B.E., Grant C.N. (2022). Cell adhesion molecules in neuroblastoma: complex roles. Therap. Potential. Front. Oncol..

[39] Feng Y., Wang Y., Zhu Z., Li W., Sussman R.T., Randall M. (2016). Differential killing of CD56-expressing cells by drug-conjugated human antibodies targeting membrane-distal and membrane-proximal non-overlapping epitopes. MAbs.

[40] Mlakar V., Jurkovic Mlakar S., Lopez G., Maris J.M., Ansari M. (2017). Gumy-Pause F. 11q deletion in neuroblastoma: a review of biological and clinical implications. Mol. Cancer.

[41] Mechtersheimer S., Gutwein P., Agmon-Levin N., Stoeck A., Oleszewski M., Riedle S. (2001). Ectodomain shedding of L1 adhesion molecule promotes cell migration by autocrine binding to integrins. J. Cell Biol..

[42] Gavert N., Conacci-Sorrell M., Gast D., Schneider A., Altevogt P., Brabletz T. (2005). L1, a novel target of β-catenin signaling, transforms cells and is expressed at the invasive front of colon cancers. J. Cell Biol..

[43] Silletti S., Yebra M., Perez B., Cirulli V., McMahon M., Montgomery A.M.P. (2004). Extracellular signal-regulated kinase (ERK)-dependent gene expression contributes to L1 cell adhesion molecule-dependent motility and invasion. J. Biol. Chem..

[44] Künkele A., Taraseviciute A., Finn L.S., Johnson A.J., Berger C., Finney O. (2017). Preclinical assessment of CD171-directed CAR T-cell adoptive therapy for childhood neuroblastoma: CE7 epitope target safety and product manufacturing feasibility. Clin. Cancer Res..

[45] Oberthuer A., Hero B., Spitz R., Berthold F., Fischer M. (2004). The tumor-associated antigen PRAME is universally expressed in high-stage neuroblastoma and associated with poor outcome. Clin. Cancer Res..

[46] Shen X., Zhao B. (2018). Efficacy of PD-1 or PD-L1 inhibitors and PD-L1 expression status in cancer: meta-analysis. BMJ.

[47] Voeller J., Sondel P.M. (2019). Advances in anti-GD2 immunotherapy for treatment of high-risk neuroblastoma. J. Pediatr. Hematol. Oncol..

[48] Pabani A., Gainor J.F. (2023). Facts and hopes: immunocytokines for cancer immunotherapy. Clin. Cancer Res..

[49] Desai A.V, Gilman A.L., Ozkaynak M.F., Naranjo A., London W.B., Tenney S.C. (2022). Outcomes following GD2-directed postconsolidation therapy for neuroblastoma after cessation of random assignment on ANBL0032: a report from the children's oncology group. J. Clin. Oncol..

[50] Yu A.L., Gilman A.L., Ozkaynak M.F., London W.B., Kreissman S.G., Chen H.X. (2010). Anti-GD2 antibody with GM-CSF, interleukin-2, and isotretinoin for neuroblastoma. N. Engl. J. Med..

[51] Ogawa M., Hori H., Ohta T., Onozato K., Miyahara M., Komada Y. (2005). Sensitivity to gemcitabine and its metabolizing enzymes in neuroblastoma. Clin. Cancer Res..

[52] Wagner-Bohn A., Paulussen M., Vieira Pinheiro J.P., Gerss J., Stoffregen C., Boos J. (2006). Phase II study of gemcitabine in children with solid tumors of mesenchymal and embryonic origin. Anticancer Drugs.

[53] Geoerger B., Chisholm J., Le Deley M.-C., Gentet J.-C., Zwaan C.M., Dias N. (2011). Phase II study of gemcitabine combined with oxaliplatin in relapsed or refractory paediatric solid malignancies: an innovative therapy for children with Cancer European Consortium Study. Eur. J. Cancer.

[54] Besançon O.G., Tytgat G.A.M., Meinsma R., Leen R., Hoebink J., Kalayda G.V (2012). Synergistic interaction between cisplatin and gemcitabine in neuroblastoma cell lines and multicellular tumor spheroids. Cancer Lett..

[55] Orienti I., Farruggia G., Nguyen F., Guan P., Calonghi N., Kolla V. (2020). Nanomicellar lenalidomide–fenretinide combination suppresses tumor growth in an MYCN amplified neuroblastoma tumor. Int. J. Nanomed..

[56] Xu Y., Sun J., Sheard M.A., Tran H.C., Wan Z., Liu W.Y. (2013). Lenalidomide overcomes suppression of human natural killer cell anti-tumor functions by neuroblastoma microenvironment-associated IL-6 and TGFβ1. Cancer Immunol. Immunother..

[57] Kretschmar C.S., Kletzel M., Murray K., Thorner P., Joshi V., Marcus R. (2004). Response to paclitaxel, topotecan, and topotecan-cyclophosphamide in children with untreated disseminated neuroblastoma treated in an upfront phase II investigational window: a pediatric oncology group study. J. Clin. Oncol..

[58] Riccardi A., Servidei T., Tornesello A., Puggioni P., Mastrangelo S., Rumi C. (1995). Cytotoxicity of paclitaxel and docetaxel in human neuroblastoma cell lines. Eur. J. Cancer.

[59] Geller J.I., Wall D., Perentesis J., Blaney S.M., Bernstein M. (2009). Pediatric oncology group study 9376. Phase I study of paclitaxel with standard dose ifosfamide in children with refractory solid tumors: a Pediatric Oncology Group study (POG 9376). Pediatr. Blood Cancer.

[60] Kroesen M., Büll C., Gielen P.R., Brok I.C., Armandari I., Wassink M. (2016). Anti-GD2 mAb and Vorinostat synergize in the treatment of neuroblastoma. Oncoimmunology.

[61] van den Bijgaart R.J.E., Kroesen M., Brok I.C., Reijnen D., Wassink M., Boon L. (2020). Anti-GD2 antibody and Vorinostat immunocombination therapy is highly effective in an aggressive orthotopic neuroblastoma model. Oncoimmunology.

[62] Phimmachanh M., Han J.Z.R., O'Donnell Y.E.I., Latham S.L., Croucher D.R (2020). Histone deacetylases and histone deacetylase inhibitors in neuroblastoma. Front. Cell Dev. Biol..

[63] Kast V., Nadernezhad A., Pette D., Gabrielyan A., Fusenig M., Honselmann K.C. (2023). A tumor microenvironment model of pancreatic cancer to elucidate responses toward immunotherapy. Adv. Healthc. Mater..

[64] Nolan J.C., Frawley T., Tighe J., Soh H., Curtin C., Piskareva O. (2020). Preclinical models for neuroblastoma: advances and challenges. Cancer Lett..

[65] Heinze A., Grebe B., Bremm M., Huenecke S., Munir T.A., Graafen L. (2019). The synergistic use of IL-15 and IL-21 for the generation of NK cells from CD3/CD19-depleted grafts improves their ex vivo expansion and cytotoxic potential against neuroblastoma: perspective for optimized immunotherapy post haploidentical stem cell. Trans. Front. Immunol..

[66] Kholosy W M., Derieppe M., van den Ham F., Ober K., Su Y., Custers L. (2021). Neuroblastoma and DIPG organoid coculture system for personalized assessment of novel anticancer immunotherapies. J. Pers. Med..

[67] Cornel A.M., Dunnebach E., Hofman D.A., Das S., Sengupta S., van den Ham F. (2022). Epigenetic modulation of neuroblastoma enhances T cell and NK cell immunogenicity by inducing a tumor-cell lineage switch. J. Immunother. Cancer.

[68] Strijker J.G.M., Pscheid R., Drent E., van der Hoek J.J.F., Koopmans B., Ober K. (2021). αβ-T cells engineered to express γδ-T cell receptors can kill neuroblastoma organoids independent of MHC-I expression. J. Pers. Med..

[69] Grunewald L., Lam T., Andersch L., Klaus A., Schwiebert S., Winkler A. (2021). A reproducible bioprinted 3D tumor model serves as a preselection tool for CAR T cell. Therapy Optimiz. Front. Immunol..

[70] Brignole C., Calarco E., Bensa V., Giusto E., Perri P., Ciampi E. (2023). Antitumor activity of the investigational B7-H3 antibody-drug conjugate, vobramitamab duocarmazine, in preclinical models of neuroblastoma. J. Immunother. Cancer.

[71] Moradpoor R., Gharebaghian A., Shahi F., Mousavi A., Salari S., Akbari M.E. (2020). Identification and validation of stage-associated PBMC biomarkers in breast cancer using MS-based proteomics. Front. Oncol..

[72] Foulds G.A., Vadakekolathu J., TMA A.-F., Nagarajan D., Reeder S., Johnson C. (2018). Immune-phenotyping and transcriptomic profiling of peripheral blood mononuclear cells from patients with breast cancer: identification of a 3 gene signature which predicts relapse of triple negative breast cancer. Front. Immunol..

[73] Prabhu S.B., Rathore D.K., Nair D., Chaudhary A., Raza S., Kanodia P., et al. Comparison of Human Neonatal and Adult Blood Leukocyte Subset Composition Phenotypes. Yu XG, editor. PLoS One [Internet]. 2016 Sep 9;11(9):e0162242. Available from: https://dx.plos.org/10.1371/journal.pone.0162242. 10.1371/journal.pone.0162242 PMC5017693 27610624

[74] Lilic D., Cant A.J., Abinun M., Calvert J.E., Spickett G.P. (1997). Cytokine production differs in children and adults. Pediatr. Res..

[75] Verhoeven B.M., Mei S., Olsen T.K., Gustafsson K., Valind A., Lindström A. (2022). The immune cell atlas of human neuroblastoma. Cell Rep. Med..

[76] Dhatchinamoorthy K., Colbert J.D., Rock K.L (2021). Cancer immune evasion through loss of MHC class I antigen presentation. Front. Immunol..

[77] Curtin C., Nolan J.C.C., Conlon R., Deneweth L., Gallagher C., Tan Y.J.J. (2018). A physiologically relevant 3D collagen-based scaffold–neuroblastoma cell system exhibits chemosensitivity similar to orthotopic xenograft models. Acta Biomater..

[78] Sidarovich V., De Mariano M., Aveic S., Pancher M., Adami V., Gatto P. (2018). A high-content screening of anti-cancer compounds suggests the multiple tyrosine kinase inhibitor ponatinib for repurposing in neuroblastoma therapy. Mol. Cancer Ther..

[79] Alonso-Nocelo M., Raimondo T.M., Vining K.H., López-López R., de la Fuente M., Mooney D.J. (2018). Matrix stiffness and tumor-associated macrophages modulate epithelial to mesenchymal transition of human adenocarcinoma cells. Biofabrication.

[80] Li T., Li Y., Zhu X., He Y., Wu Y., Ying T. (2023). Artificial intelligence in cancer immunotherapy: applications in neoantigen recognition, antibody design and immunotherapy response prediction. Semin. Cancer Biol..

